# EMQN: Recommendations for genetic testing in inherited cardiomyopathies and arrhythmias

**DOI:** 10.1038/s41431-023-01421-w

**Published:** 2023-07-13

**Authors:** Jesse B. Hayesmoore, Zahurul A. Bhuiyan, Domenico A. Coviello, Desirée du Sart, Matthew Edwards, Maria Iascone, Deborah J. Morris-Rosendahl, Katie Sheils, Marjon van Slegtenhorst, Kate L. Thomson

**Affiliations:** 1 Oxford Regional Genetics Laboratories, Oxford University Hospitals NHS Foundation Trust, Oxford, UK; 2 Division of Genetic Medicine, Centre Hospitalier Universitaire Vaudois (CHUV), Lausanne, Switzerland; 3 Laboratory of Human Genetics, IRCCS Istituto Giannina Gaslini, Genoa, Italy; 4 Biological Sciences and Genomics, Monash University, Melbourne, VIC, Australia; 5 Clinical Genetics and Genomics Laboratory, Royal Brompton and Harefield Hospitals, Guy’s and St. Thomas’ NHS Foundation Trust, London, UK; 6 Laboratorio di Genetica Medica, ASST Papa Giovanni XXIII, Bergamo, Italy; 7 EMQN, Manchester, UK; 8 Clinical Genetics, Erasmus Medical Center, Rotterdam, The Netherlands

**Keywords:** Genetic testing, Cardiovascular diseases

## Abstract

Inherited cardiomyopathies and arrhythmias (ICAs) are a prevalent and clinically heterogeneous group of genetic disorders that are associated with increased risk of sudden cardiac death and heart failure. Making a genetic diagnosis can inform the management of patients and their at-risk relatives and, as such, molecular genetic testing is now considered an integral component of the clinical care pathway. However, ICAs are characterised by high genetic and allelic heterogeneity, incomplete / age-related penetrance, and variable expressivity. Therefore, despite our improved understanding of the genetic basis of these conditions, and significant technological advances over the past two decades, identifying and recognising the causative genotype remains challenging. As clinical genetic testing for ICAs becomes more widely available, it is increasingly important for clinical laboratories to consolidate existing knowledge and experience to inform and improve future practice. These recommendations have been compiled to help clinical laboratories navigate the challenges of ICAs and thereby facilitate best practice and consistency in genetic test provision for this group of disorders. General recommendations on internal and external quality control, referral, analysis, result interpretation, and reporting are described. Also included are appendices that provide specific information pertinent to genetic testing for hypertrophic, dilated, and arrhythmogenic right ventricular cardiomyopathies, long QT syndrome, Brugada syndrome, and catecholaminergic polymorphic ventricular tachycardia.

## Introduction

It has been almost two decades since the first molecular genetic tests for inherited cardiomyopathies and arrhythmias (ICAs) were translated into the clinical laboratory; in many countries, these services are now considered a routine component of clinical care [1–3]. Over this period, technological developments have enabled laboratories to improve and expand test services. Consequently, there has been a huge increase in the number of clinical laboratories providing testing and a wide range of massively parallel sequencing (MPS) gene panel tests are now available.

However, as more genetic data becomes available from case cohorts, and from large-scale population-based cohorts, we are gaining a better understanding of the genetic basis of these conditions and the challenges associated with genetic testing in a clinical setting. The recent EHRA/HRS/APHRS/LAHRS consensus statement on the state of genetic testing for cardiac diseases provides a comprehensive overview of the principles of genetic disease and genetic testing, the clinical characteristics of the common inherited cardiac conditions, and guidance on the clinical utility of testing in these conditions [4]. Here, we focus on the main challenges in genetic testing for ICAs and provide recommendations for clinical testing laboratories to facilitate best practice and encourage consistency in test provision.

These guidelines focus on the most common inherited cardiomyopathies and arrhythmias. Recommendations common to these conditions are presented below. This general guidance is supplemented by disease-specific appendices which provide relevant clinical and genetic information for each condition (Table 1). Disease-specific appendices do not extend to rare conditions/phenotypes where the evidence base relating to the genetic basis is still evolving, clinical genetic testing is less well-established, and ClinGen disease-gene curations are not yet available (e.g., non-compaction cardiomyopathy, restrictive cardiomyopathy) [4].

**Table 1:** Disease-specific appendices.

Inherited cardiac condition	Disease specific information
Hypertrophic cardiomyopathy (HCM)	Appendix 1
Dilated cardiomyopathy (DCM)	Appendix 2
Arrhythmogenic right ventricular cardiomyopathy (ARVC)	Appendix 3
Long QT syndrome (LQTS)	Appendix 4
Brugada syndrome (BrS)	Appendix 5
Catecholaminergic polymorphic ventricular tachycardia (CPVT)	Appendix 6

See Supplementary Information.

### Inherited cardiomyopathies and arrhythmias

Inherited cardiomyopathies and arrhythmias encompass a group of clinically and genetically heterogeneous conditions that affect different aspects of heart function [1–3]. Due to the risk of sudden cardiac death (SCD) and heart failure associated with these conditions, early diagnosis and management are critical [1–4].

### Clinical features of common inherited cardiomyopathies and arrhythmias

The key clinical features, prevalence estimates, and clinical guidelines relevant to each ICA are provided in the disease-specific appendices. The clinical features and symptoms of these disorders can be variable, even within families, and can appear at any age [1–3]. Another complicating factor, common to all ICAs, is incomplete penetrance, whereby some individuals carrying a known disease-causing variant do not appear to develop the condition even at an advanced age, and phenotypic variability within families, even with the same genetic cause, is extensive [1–3].

### Genetic basis of common inherited cardiac conditions

The genetic characteristics of each ICA, including the definitive genes; classes of variants considered to be pathogenic; mode of inheritance; and genotype-phenotype characteristics; are provided in the disease-specific appendices. Most of these conditions are caused by monoallelic variants in single genes, and inherited in an autosomal dominant manner, often with incomplete and age-related penetrance [1–4]. Autosomal recessive forms (caused by biallelic variants in a single gene), or digenic forms (caused by monoallelic variants in two genes) are also described, and, rarely, variants in X-linked and mitochondrial genes (note: variants in mitochondrial genes are not part of the scope in this document) [4]. The appendix for HCM also includes genes in which variants cause important phenocopy disorders that may be misdiagnosed as HCM. In most of the ICA genes, pathogenic variants cause primary cardiomyopathy or arrhythmia; however, a small number of genes are associated with syndromic disorders. Recurrent and founder disease-causing variants have been described in some genes [5–7]; however, many variants appear to be novel, or private to a family. While there are several well-established ICA genes, there are also many genes where the genotype/phenotype relationship is less definitive [8–13].

### Genetic testing

The clinical utility of genetic testing for individuals with ICAs has been widely reported and genetic testing is now considered an integral component of the diagnosis and management of affected individuals and their at-risk relatives [1–3]. Genetic testing should be initially performed in the index case (the first affected individual in a family referred for clinical screening because of symptoms suggestive of the disorder), or the most severely affected individual in a family, to determine if a variant in one of the known genes has contributed to their phenotype [1–4]. If a disease-causing variant is identified in the index case, genetic testing can be used to definitively identify relatives, who may be at increased risk of developing the disease and of sudden death, and release genotype-negative individuals from clinical screening [1–4]. However, the clinical and genetic heterogeneity of these conditions present challenges in terms of test strategy and results interpretation [4, 14].

## Methods

Eight representatives from five centres across Europe were invited to share their expertise and experience in genetic testing for inherited cardiomyopathies and arrhythmias. The representatives met in person on 11th February 2020 to discuss the writing strategy and thereafter conducted virtual meetings over regular intervals between March 2020 and October 2021 to draft the guidelines through group consensus.

The following points were discussed:

- Referral criteria
- Testing strategy
- Genes tested
- Methodology
- Variant detection rate
- Interpretation of results
- Tools/resources used to investigate pathogenicity
- Classification of variants
- Reporting

The main challenges and barriers to testing were discussed and different approaches to practice explored. Following the creation of the draft guidelines, the document was made available through EMQN to a community of 85 participating laboratories in the EMQN-organised external quality assessment schemes for Cardiac Arrhythmias and Hypertrophic Cardiomyopathies. The community consultation period was held between 23rd June and 25th July 2022. Based on the representative discussions, as well as feedback collected and evaluated during the EMQN community consultation, final consensus recommendations for genetic testing in ICA were defined. In addition to these general recommendations, it was decided that brief, disease-specific summaries would be provided for individual indications (Appendices 1–6).

## Recommendations

These guidelines have been produced to assist laboratories in developing a consistent ‘best’ practice for the following:

- Internal and external quality control
- Referral
- Analysis
- Results interpretation
- Reporting

These recommendations are based on data from the literature and from participating clinical laboratories.

### Internal and external quality control

It is recommended that all laboratories offering molecular genetic testing for cardiac conditions follow established good laboratory practice, as documented for example in Guidelines for Quality Assurance in Molecular Genetic Testing [15], published by the Organisation for Economic Co-operation and Development and MM09 - Nucleic Acid Sequencing Methods in Diagnostic Laboratory Medicine, 2nd Edition from the Clinical and Laboratory Standards Institute (CLSI).

In addition to following such guidelines, a laboratory should ideally demonstrate that it complies with internationally recognised standards for laboratory testing (e.g., ISO standards 15189: 2012 Medical laboratories – requirements for quality and competence), by achieving formal accreditation with a member organisation of the International Laboratory Accreditation Cooperation (ILAC) or equivalent national accreditation body.

All tests should be validated/verified in individual laboratories prior to implementation; it is not acceptable to rely on the validation of a test by another laboratory since that does not guarantee that it will perform accurately and reliably in all labs. A series of control samples representing all variant types should therefore be collected by each laboratory to facilitate test validation/verification, and exchange of samples between laboratories is encouraged to allow this. It is also recommended to use reference samples in the validation of NGS assays [16]. External quality assessment (EQA) schemes provide further validation of testing procedures and methods, and laboratories should participate annually in appropriate EQA schemes for cardiac genetic testing including independent evaluation of testing and of reporting. Annual participation in a technical EQA evaluating NGS performance is also recommended. If this is not possible, inter-laboratory exchange of samples should be arranged to compare and validate test results.

### Referral

A multidisciplinary approach, involving close communication between Cardiology, Clinical Genetics and the testing laboratory is recommended throughout the referral process. Clinical diagnosis should be made or discussed within an experienced cardiology department; guidelines relating to the clinical diagnosis and management of individuals with inherited conditions have previously been published and will not be reiterated here [1–4]. Genetic counselling by trained healthcare professionals with specialised knowledge of these cardiac conditions is recommended for individuals considering genetic testing, in particular for relatives of individuals diagnosed with an inherited cardiac condition [17].

In cases of unexplained sudden death, referrals may be received from Pathology Departments; again, a multidisciplinary approach involving close communication between Pathology, Clinical Genetics, and the testing laboratory, will facilitate this process and ensure that the family receives appropriate counselling prior to testing and that the most appropriate samples are taken for genetic analysis.

#### Information provided with referral

In addition to basic patient demographic data (i.e., age, sex, ethnicity), clinical information should be provided with each referral to ensure the laboratory undertakes the most appropriate analysis. Minimally, this should include the headline phenotype (e.g., ‘hypertrophic cardiomyopathy’, or ‘long QT syndrome’), age at diagnosis (if different from age at referral), and information on family history (ideally a pedigree showing familial relationships, sex, and clinical status).

More detailed clinical information may be required when referring atypical or unusual cases (e.g., early age of onset, severe phenotype, extra-cardiac features, mixed cardiac phenotypes within family) as alternative test strategies may be considered. For example, additional genes may be added to the routine test panel, or alternative test panels may be initiated to cover a broader range of phenotypes or inheritance patterns (autosomal recessive, X-linked, or mitochondrial). Details on the genes and / or genetic disorders which may be considered as part of the differential diagnoses for each ICA are provided in the disease-specific appendices.

### Analysis

The core genes recommended for inclusion in test panels for each ICA are listed in the disease-specific appendices. These lists take into consideration existing ClinGen gene-disease clinical validity curation publications [8–13]. Testing of additional genes may be appropriate; however, testing is only recommended for genes where there is robust empirical evidence supporting a gene-disease relationship. Ongoing gene curation efforts will inform the core content of future clinical test panels for each ICA.

#### Evaluating genes for inclusion in test panels

The number of genes linked to ICAs has increased considerably in recent years and the increased capacity offered by high throughput sequencing methodologies means that these ‘new’ genes are readily incorporated into test panels. However, for many of the recently implicated genes there is little evidence to support a causal role (e.g., small case numbers, limited or no segregation studies, no functional data), as such it is difficult to interpret the clinical significance of rare variants detected in these genes, and many are reported as VUS [18, 19]. To avoid high numbers of inconclusive, clinically un-actionable results, it is recommended that analyses focus on genes with a definitive gene-disease relationship.

The Clinical Genome Resource (ClinGen) gene curation working groups have an active program of gene ICA gene curation, therefore, information supporting the clinical validity of a gene-disease pair may be available on the Clinical Genome Resource (ClinGen) website (https://clinicalgenome.org/working-groups/gene-curation/). Where this information is not available, laboratories should curate the available evidence prior to considering a gene for inclusion in a test panel. The ClinGen Gene Curation working group has created a framework to facilitate and standardise gene curation and provides resources that may be used by clinical laboratories for curation activities [20].

#### Selection of test panel

To minimise the risk of identifying variants of uncertain clinical significance, for straightforward cases with ‘typical’ phenotype, we would caution against broad, overly inclusive genetic analysis. However, in cases where the clinical phenotype in an individual and/or their extended family is unclear, where multiple phenotypes exist in one family, or where there is atypical presentation (early age of onset, severe phenotype, extra cardiac features), it may be appropriate to use an extended test panel incorporating genes from a broader range of ICA phenotypes.

#### Individuals with more than one variant

Despite uncertainty in the frequency of cases with more than one pathogenic variant, it is acknowledged that cases of compound / double heterozygosity do occur [21]; therefore, complete analysis of key genes is warranted, particularly in cases with early age of onset and/or more severe presentation.

#### Test methodology

The majority of pathogenic variants detected in ICA genes are single nucleotide substitutions and small insertions/deletions [18, 19, 22]. Therefore, analysis techniques should be able to detect these types of variants with high sensitivity and specificity. In some genes, larger insertions/deletions involving one or more exons of a gene have been reported [23–25]. Therefore, copy number variant (CNV) analyses are recommended for some genes (e.g., computational methods to detect CNVs in sequencing data or multiplex-ligation dependent probe amplification [MLPA] analysis). Testing for intronic variants, beyond the canonical splice acceptor/donor sites, may be appropriate for some genes [26–29]; frequently detected pathogenic variants that lie within deeper intronic regions beyond canonical splice sites and adjacent sequences are listed in the disease-specific appendices.

#### Considerations for variant calling and annotation

An important consideration for laboratories undertaking genetic testing is the choice of reference transcript/s used for variant calling and annotation. Where possible, to ensure consistency and continuity, and to facilitate variant sharing, laboratories should use the transcripts selected by the Matched Annotation from the NCBI and EMBL-EBI (MANE) project [30] for variant calling and annotation. However, additional transcripts may be required to ensure that the isoforms predominantly expressed in cardiac tissue are represented. Information on the genome build and reference transcript/s used (including version number), should be documented. The use of genome build GRCh38 / hg38 is recommended. The commonly used transcripts for the key genes are listed in the disease-specific appendices. For new and/or novel genes, careful consideration should be given to transcript selection to minimise the risk of false positive or negative variant annotation.

### Results interpretation

We recommend that variant assessment and classification is carried out according to the published American College of Medical Genetics (ACMG)/Association for Molecular Pathology (AMP) guidelines [31], taking into consideration the ClinGen adapted guidelines for the *MYH7* gene [32], and additional ClinGen guidance relating to specific ACMG/AMP criteria [33, 34]. It is important to note that guidance on variant interpretation is continually evolving, and additional disease- and/or gene-specific guidance is likely to become available in the future. Periodic review and re-evaluation of previously described variants should be considered to ensure that classification is based on contemporary evidence and interpretation guidelines [35]. General points to consider when evaluating variants in ICA genes are highlighted below.

#### Interpretation of truncating variants

Existing guidelines recognise that the predicted protein-truncating nature of certain types of variants (e.g., nonsense or frameshift variants, exon-scale deletions, or variants that affect canonical [+/− 1 or 2] splice sites or initiation codons) can be considered ‘strong’ or ‘very strong’, evidence for a pathogenic classification [33]. The genes in which truncating variants are known to play a causal role in disease are listed in the disease-specific appendices. This should not be considered an exhaustive list, and furthermore, non-truncating variants detected in these genes cannot be assumed to be benign. Importantly, even in these genes, the classification of a truncating variant as pathogenic should not be automatic, but only made after consideration of important caveats. Existing guidelines recommend caution regarding novel truncating variants located more 3’ than any truncating variant previously established as pathogenic in the literature [33] In particular, caution should be applied whenever the novel stop codon is predicted to occur in the final exon or within 50 bp upstream (5’) of the final splice junction, as stop signals in this region may not elicit nonsense-mediated mRNA decay (i.e., functional or partially functional protein may still be expressed) [33].

The mRNA isoform or exon that the variant is predicted to affect should also be considered. A truncating variant that is predicted to primarily affect an isoform or exon that is not significantly expressed in cardiac tissue is unlikely to be pathogenic. The consideration of alternative splice forms also extends to missense and other types of in-frame variants. Any variant that is predicted to exclusively affect an mRNA splice form that is known not to be expressed in cardiac tissue should be considered unlikely pathogenic.

Truncating variants in genes where there is no strong evidence to support the causality of this variant type should not be automatically dismissed as benign. For example, splice variants may cause in-frame deletions or insertions at RNA / protein levels, while frameshift variants in the terminal exon may be expected to introduce a series of missense changes in the protein. Hence, although not obviously pathogenic, these types of variants may still have pathogenic potential.

#### RNA studies

The putative effect of variants on splicing can be investigated or verified by studies of RNA extracted from tissue or by in vitro splicing tests, such as minigene assays [36]. Demonstration of an effect on splicing that is not seen in at least one matched control sample can be considered strong evidence for pathogenicity if studies are undertaken on RNA extracted from cardiac tissue. However, it is recognised that cardiac tissue is rarely available for analysis. For some genes, it may be possible to perform RNA studies using more readily available tissue, such as peripheral blood. Since splice patterns in blood may not necessarily represent those in cardiac tissue, an effect should be interpreted with a degree of caution. If non-cardiac tissue is analysed, it is important to consider existing knowledge regarding the tissue specific splicing patterns of the relevant exon.

#### Functional studies

Functional studies can provide important information that can help to distinguish truly pathogenic variants from benign linked markers. Although few diagnostic laboratories have the resources to undertake these studies, many functional assessments of variants in ICA genes can be found in the literature. Literature reports of functional studies should be interpreted with caution and – where the knowledge exists – with a clear understanding of the normal functions of the relevant proteins. For example, since HCM is primarily a disease of the sarcomere, readers should at least have a basic understanding of sarcomere structure and of the roles played by relevant proteins, ATP hydrolysis, and Ca^2+^ ions in the sliding filament theory of muscle contraction. It should also be borne in mind that many functional assays are highly sensitive to experimental conditions. This makes it very difficult for scientists with no experience of the assay to appreciate parameters that may bias the results.

When evaluating functional studies, it is important to consider how closely the experiment or measured effect might reflect the situation in human cardiac tissue or cardiomyocytes [34]. For example, owing to differences in sarcomeric protein composition between murine and human cardiac ventricular tissue, murine models may not precisely recapitulate the phenotype in humans (e.g., α-myosin is the predominant cardiac isoform in mice, versus β-myosin in humans) [37].

Additionally, the directionality of any reported effects should be borne in mind when interpreting functional studies. For example, a significant body of evidence suggests that pathogenic HCM variants generally cause an increase in calcium sensitivity, consistent with the diastolic dysfunction characteristic of HCM. The opposite effect (i.e., decreased calcium sensitivity) is more often observed with variants related to DCM [38].

Recommendations for evaluation and weighting of functional evidence have been published by the ClinGen Sequence variant interpretation working group [34]. However, it is acknowledged that few, if any, published functional studies investigating ICA gene variants would meet the stringent criteria proposed in this document.

#### Use of normal control or population cohort variant frequency data

The assessment of variant frequency in healthy or random control cohorts, or in population-based cohorts, is recognised as a useful means to evaluate potential pathogenicity. Current guidelines state that a control allele frequency of >5% is sufficient evidence for a benign classification [31]. They also state that for early-onset, fully penetrant, dominant conditions, the detection of a variant in just one healthy adult individual can be considered strong evidence for a benign interpretation. However, ICAs are rarely early-onset, frequently non-penetrant or undiagnosed, and do not generally affect reproductive success; hence, for most ICAs, the detection of pathogenic variants in control cohorts is not unexpected. Therefore, when evaluating variant frequency in control cohorts, the primary aim is not necessarily to demonstrate absence of the variant, but instead to demonstrate that the variant is not more frequent in controls or the general population than would be expected given conservative estimates for the disease prevalence, penetrance, and genetic heterogeneity.

In the recent past, the non-detection of a variant in just a few hundred control individuals was used as evidence for pathogenicity. However, a cohort of this magnitude is severely under-powered. For example, the non-detection of a variant in 300 control individuals provides 95% confidence that that the variant is not detectable in only 1 in 100 members of the general population. Clearly, for conditions with prevalence of less than 1 in 500 individuals, and for which the vast majority of pathogenic variants account for fewer than 1% of probands (if not ‘private’ to individual families), much larger cohorts of controls (e.g., thousands to tens of thousands) are required for meaningful inferences to be drawn.

Since few diagnostic genetics laboratories have access to large normal control cohorts, the assessment of variant frequency in publicly available population databases is considered an appropriate alternative. The Genome Aggregation Database (gnomAD, www.gnomad.broadinstitute.org) [39] is widely used for this purpose. Currently, this resource includes sequence data for >200,000 unrelated individuals sequenced as part of various disease-specific and population genetic studies. While none were selected for ICA phenotypes, it should be borne in mind that individuals with an inherited cardiomyopathy or arrhythmia are likely to be included in this dataset at a frequency assumed to approximate population prevalence. Accordingly, these databases include known pathogenic variants in some ICA genes and are likely to also include currently unknown pathogenic variants. For this reason, small allele counts should not be over-interpreted as evidence for an unlikely pathogenic classification. As individuals affected by severe paediatric disease have been removed from the gnomAD database, this dataset provides a useful reference when assessing allele frequencies for variants in genes that cause severe infant onset ICAs (e.g., *CALM* genes [40]).

The effective use of population-based cohorts is dependent on the setting of a disease- or gene-specific allele frequency threshold. Gene-specific minor allele frequency (MAF) thresholds are specified in the existing *MYH7* guidelines [32]. These MAF thresholds calculations were based on the frequency of one of the most common known pathogenic variants amongst the inherited cardiac conditions covered by these guidelines (*MYBPC3*, p.(Arg502Trp) [18, 19]). As such, they are expected to be sufficiently conservative for assessment of variants in the other inherited ICA genes in the context of dominant inheritance. However, these population MAF thresholds are not applicable when assessing variants that display markedly reduced or incomplete penetrance (e.g., *KCNQ1* p.(Arg518*) [41]), or where there is a possibility of recessive inheritance (e.g., *CASQ2* [42] or *MYL2* [43]).

Additionally, although many pathogenic variants are rare, if not private, pathogenic founder variants with relatively high frequency have been described in some populations [5–7]. Clearly, the above recommendations do not apply to founder variants that are well-established to be pathogenic. The possibility of yet-to-be-identified founder variants should always be borne in mind, especially with regards to probands from populations known to have proliferated from small or isolated settlements. In addition to founder variants, the existence of population-specific benign polymorphisms should also be borne in mind. For this reason, zero or low cohort frequencies should only be used as evidence for pathogenicity if the ancestry of the proband is well-represented in the control cohort.

#### Segregation studies

Segregation studies are a useful means to gather evidence in support of either a pathogenic or a benign classification. However, it is important to remember that these studies only provide evidence for or against linkage of the locus with the phenotype, not the pathogenicity of the variant. Therefore, segregation studies should ideally only be undertaken in families of probands in whom all relevant regions of the gene have been analysed and should be approached with extreme caution in consanguineous pedigrees. Demonstration of segregation of a variant with affected status in multiple unrelated families provides much greater confidence of the pathogenic status of a specific variant. General guidance on the weighting of segregation data as evidence for or against pathogenicity has been provided elsewhere; following this guidance is recommended [32, 44].

The utility of segregation studies can be limited by confounding factors, several of which are particularly relevant to ICAs. For example, many causal variants are incompletely penetrant or have age-related penetrance; hence, the detection of a variant in unaffected (especially young) family members cannot be used as evidence against pathogenicity [14]. Conversely, due to the relatively high prevalence of non-genetic phenocopies (e.g., hypertensive hypertrophy, athlete’s heart, etc), or non-specific symptoms (e.g., syncope, palpitations), the non-detection of a variant in an apparently affected family member should only be used as evidence against pathogenicity if the clinical diagnosis in those individuals is certain.

For these reasons, it is highly recommended that segregation studies are only undertaken in family members who have undergone thorough cardiological investigations [4, 14]. Also, because the result will have no clinical predictive value, testing for variants of uncertain significance in unaffected family members is not recommended [14, 17]. Ideally, the clinical status of family members with ‘borderline’ or ‘possibly affected’ cardiac phenotypes should be clarified prior to testing, as knowledge of the test result could bias the diagnosis. If family members with uncertain clinical status are tested, the results should not be used as evidence for or against pathogenicity until clinical status has been clarified.

Molecular screening of large gene panels has meant that the detection of multiple potentially pathogenic variants is quite a frequent occurrence [18, 19, 22]. This phenomenon complicates segregation studies. Generally, for dominant cardiomyopathies and arrhythmias, the non-detection of a variant in an affected family member who has another variant cannot be used as evidence against pathogenicity of the non-detected variant [32]. In contrast, detection of >1 variant in an affected family member should not generally be used as evidence for pathogenicity of either variant. However, the correlation of >1 variant in family members with a more severe or earlier-onset phenotype as compared with affected members with just one variant can provide some evidence for pathogenicity of both variants [21]. Nonetheless, caution should be applied when using this logic, as pathogenic variants can exhibit significant variable expressivity (e.g., some pathogenic *TPM1* missense variants are associated with extreme intrafamilial variation in both age at onset and severity of cardiomyopathy [45]). The clearest evidence for or against pathogenicity of either variant is obtained from affected family members that either have neither or just one variant.

#### De novo variants

Probands with inherited cardiomyopathies and arrhythmias often have no clinically affected relatives. Assuming a genetic aetiology, in many of the ICAs, non-penetrance is a common explanation; however, an alternative is that the phenotype in the index case is due to a de novo variant. Since these conditions usually have a minimal effect on reproductive success, in the majority of ICA genes, de novo variants are generally not a frequent aetiologic mechanism [19]. However, they are found at a higher rate in genes which cause paediatric onset disorders (e.g., *CALM1*-related LQTS [40] and *RYR2-*related CPVT [46]) and in cases with early-onset presentation in the absence of a family history [19, 47].

### Reporting

It is appreciated that reporting guidelines are influenced by local policy/practice and vary between laboratories and countries. There are a few published guidelines [48, 49]; readers should refer to these, and to local guidance, for information relating to best practice in reporting of genetic test results. Ideally, results should be reported to healthcare professionals with specialised knowledge of genetics and inherited cardiac conditions, to ensure that the clinical implications of any findings are considered for the index case and the family, and to facilitate cascade testing of at-risk relatives where appropriate [4, 14, 17]. If results are being reported to a non-specialist, it may be appropriate to include a statement in the report recommending referral to a specialist genetics centre.

Individuals with pathogenic variants in certain genes may be at risk of developing extra-cardiac clinical features (refer to disease-specific appendices for further details). Detection of pathogenic variants in these genes is also likely to inform the clinical management of the patient e.g., individuals with Fabry disease and a pathogenic *GLA* variant may be offered life-altering enzyme replacement therapy [50]. Therefore, when reporting the detection of a pathogenic variant in these genes or conditions, it is important to communicate these risks to the relevant specialist within the clinical report; reference to additional testing or clinical follow-up is also important.

Additionally, it may be helpful to confer with the referring clinician for further information prior to reporting as that may allow for more accurate variant classification, e.g., details of additional clinical or physical features, or the results of additional biochemical tests or referrals to other specialities other than cardiology.

#### Family/cascade testing

Where a ‘pathogenic’ variant has been identified in a proband, molecular testing may be offered to extended family members; the results of this analysis may be used to guide clinical management [4, 14]. More caution should be applied when dealing with variants classified as ‘likely to be pathogenic’. Genetic testing in other clinically confirmed phenotype-positive family members is recommended prior to considering predictive genetic testing in phenotype-negative relatives. Molecular testing of phenotype-negative family members is not recommended for ‘variants of uncertain significance (VUS)’, with the exception of parental testing to determine whether a variant has arisen de novo. Molecular genetic testing may be considered in clinically confirmed phenotype-positive family members; demonstration of segregation of a variant in other affected family members may provide additional evidence for pathogenicity. In some instances, it may be appropriate to test DNA from stored tissue of a deceased relative. Due to the increased risk of a false-negative result when testing DNA extracted from paraffin-embedded material, non-detection of a familial variant in this sample type should be interpreted with caution, especially when used for guiding cascade genetic testing of other relatives.

### Data sharing

To facilitate and improve results interpretation laboratories should aim to submit variant information (including headline phenotype, a summary of the evidence used to make variant classification, segregation data and the date of interpretation) to a curated locus specific database (e.g., ClinVar [https://www.ncbi.nlm.nih.gov/clinvar/] or Decipher [https://decipher.sanger.ac.uk/]). It is also important to update variant database entries as new evidence becomes available.

## Conclusion

This document reflects the consensus opinion of representatives of the writing group and EMQN scheme participants. Guidelines and expert opinions published in the literature have also been considered. These recommendations are intended to provide clinical laboratories with a practical approach to the analysis, interpretation, and reporting of referrals for genetic testing in inherited cardiomyopathies and arrhythmias.

## Supplementary information

Supplementary Appendices

## References

[1] Priori SG, Wilde AA, Horie M, Cho Y, Behr ER, Berul C (2013). HRS/EHRA/APHRS expert consensus statement on the diagnosis and management of patients with inherited primary arrhythmia syndromes: document endorsed by HRS, EHRA, and APHRS in May 2013 and by ACCF, AHA, PACES, and AEPC in June 2013. Heart Rhythm.

[2] Towbin JA, McKenna WJ, Abrams DJ, Ackerman MJ, Calkins H, Darrieux FCC (2019). 2019 HRS expert consensus statement on evaluation, risk stratification, and management of arrhythmogenic cardiomyopathy. Heart Rhythm.

[3] Ommen SR, Mital S, Burke MA, Day SM, Deswal A, Elliott P (2020). 2020 AHA/ACC guideline for the diagnosis and treatment of patients with hypertrophic cardiomyopathy: a report of the american college of cardiology/american heart association joint committee on clinical practice guidelines. J Am Coll Cardiol.

[4] Wilde AAM, Semsarian C, Márquez MF, Sepehri Shamloo A, Ackerman MJ, Ashley EA et al. European Heart Rhythm Association (EHRA)/Heart Rhythm Society (HRS)/Asia Pacific Heart Rhythm Society (APHRS)/Latin American Heart Rhythm Society (LAHRS) Expert Consensus Statement on the State of Genetic Testing for Cardiac Diseases. Heart Rhythm 2022. 10.1016/J.HRTHM.2022.03.1225. 10.1016/j.hrthm.2022.03.1225 35390533

[5] Marino TC, Maranda B, Leblanc J, Pratte A, Barabas M, Dupéré A (2017). Novel founder mutation in French-Canadian families with Naxos disease. Clin Genet.

[6] van der Zwaag PA, Cox MGPJ, van der Werf C, Wiesfeld ACP, Jongbloed JDH, Dooijes D (2010). Recurrent and founder mutations in the Netherlands: Plakophilin-2 p.Arg79X mutation causing arrhythmogenic right ventricular cardiomyopathy/dysplasia. Neth Heart J.

[7] van den Wijngaard A, Volders P, van Tintelen JP, Jongbloed JDH, van den Berg MP, Lekanne Deprez RH (2011). Recurrent and founder mutations in the Netherlands: cardiac Troponin I (TNNI3) gene mutations as a cause of severe forms of hypertrophic and restrictive cardiomyopathy. Neth Heart J.

[8] Ingles J, Goldstein J, Thaxton C, Caleshu C, Corty EW, Crowley SB (2019). Evaluating the clinical validity of hypertrophic cardiomyopathy genes. Circ Genom Precis Med.

[9] Walsh R, Adler A, Amin AS, Abiusi E, Care M, Bikker H (2022). Evaluation of gene validity for CPVT and short QT syndrome in sudden arrhythmic death. Eur Heart J.

[10] Hosseini SM, Kim R, Udupa S, Costain G, Jobling R, Liston E (2018). Reappraisal of reported genes for sudden arrhythmic death. Circulation.

[11] James CA, Jongbloed JDH, Hershberger RE, Morales A, Judge DP, Syrris P (2021). International evidence based reappraisal of genes associated with arrhythmogenic right ventricular cardiomyopathy using the clinical genome resource framework. Circ Genom Precis Med.

[12] Adler A, Novelli V, Amin AS, Abiusi E, Care M, Nannenberg EA, et al. An International, Multicentered, Evidence-Based Reappraisal of Genes Reported to Cause Congenital Long QT Syndrome. Circulation 2020;141:418–28. 10.1161/CIRCULATIONAHA.119.043132 PMC7017940 31983240

[13] Jordan E, Peterson L, Ai T, Asatryan B, Bronicki L, Brown E (2021). Evidence-based assessment of genes in dilated cardiomyopathy. Circulation.

[14] Arbustini E, Behr ER, Carrier L, van Duijn C, Evans P, Favalli V, et al. Interpretation and actionability of genetic variants in cardiomyopathies: a position statement from the European Society of Cardiology Council on cardiovascular genomics. Eur Heart J. 2022. 10.1093/EURHEARTJ/EHAB895. 10.1093/eurheartj/ehab895 35089333

[15] Organisation for economic co-operation and development (OECD). Guidelines for quality assurance in molecular genetic testing. 2007 https://www.oecd.org/science/emerging-tech/38839788.pdf (accessed 28 Mar2022).

[16] Hardwick SA, Deveson IW, Mercer TR (2017). Reference standards for next-generation sequencing. Nat Rev Genet.

[17] Charron P, Arad M, Arbustini E, Basso C, Bilinska Z, Elliott P (2010). Genetic counselling and testing in cardiomyopathies: a position statement of the European Society of Cardiology Working Group on Myocardial and Pericardial Diseases. Eur Heart J.

[18] Walsh R, Thomson KL, Ware JS, Funke BH, Woodley J, McGuire KJ (2017). Reassessment of Mendelian gene pathogenicity using 7,855 cardiomyopathy cases and 60,706 reference samples. Genet Med.

[19] Alfares AA, Kelly MA, McDermott G, Funke BH, Lebo MS, Baxter SB (2015). Results of clinical genetic testing of 2,912 probands with hypertrophic cardiomyopathy: expanded panels offer limited additional sensitivity. Genet Med.

[20] DiStefano MT, Goehringer S, Babb L, Alkuraya FS, Amberger J, Amin M (2022). The Gene Curation Coalition: A global effort to harmonize gene–disease evidence resources. Genet Med.

[21] Burns C, Bagnall RD, Lam L, Semsarian C, Ingles J. Multiple Gene Variants in Hypertrophic Cardiomyopathy in the Era of Next-Generation Sequencing. Circ Cardiovasc Genet. 2017; 10. 10.1161/CIRCGENETICS.116.001666. 10.1161/CIRCGENETICS.116.001666 28790153

[22] Walsh R, Lahrouchi N, Tadros R, Kyndt F, Glinge C, Postema PG (2021). Enhancing rare variant interpretation in inherited arrhythmias through quantitative analysis of consortium disease cohorts and population controls. Genet Med.

[23] Mates J, Mademont-Soler I, Del Olmo B, Ferrer-Costa C, Coll M, Pérez-Serra A (2018). Role of copy number variants in sudden cardiac death and related diseases: genetic analysis and translation into clinical practice. Eur J Hum Genet.

[24] Lopes LR, Murphy C, Syrris P, Dalageorgou C, McKenna WJ, Elliott PM (2015). Use of high-throughput targeted exome-sequencing to screen for copy number variation in hypertrophic cardiomyopathy. Eur J Med Genet.

[25] Ceyhan-Birsoy O, Pugh TJ, Bowser MJ, Hynes E, Frisella AL, Mahanta LM (2015). Next generation sequencing-based copy number analysis reveals low prevalence of deletions and duplications in 46 genes associated with genetic cardiomyopathies. Mol Genet Genom Med.

[26] Clemens DJ, Tester DJ, Marty I, Ackerman MJ (2020). Phenotype-guided whole genome analysis in a patient with genetically elusive long QT syndrome yields a novel TRDN-encoded triadin pathogenetic substrate for triadin knockout syndrome and reveals a novel primate-specific cardiac TRDN transcript. Heart Rhythm.

[27] Lopes LR, Barbosa P, Torrado M, Quinn E, Merino A, Ochoa JP et al. Cryptic Splice-Altering Variants in MYBPC3 Are a Prevalent Cause of Hypertrophic Cardiomyopathy. Circ Genom Precis Med. 2020; 13. 10.1161/CIRCGEN.120.002905. 10.1161/CIRCGEN.120.002905 32396390

[28] Mendes De Almeida R, Tavares J, Martins S, Carvalho T, Enguita FJ, Brito D, et al. Whole gene sequencing identifies deep-intronic variants with potential functional impact in patients with hypertrophic cardiomyopathy. PLoS One. 2017; 12. 10.1371/JOURNAL.PONE.0182946. 10.1371/journal.pone.0182946 PMC5552324 28797094

[29] Harper AR, Bowman M, Hayesmoore JBG, Sage H, Salatino S, Blair E, et al. Reevaluation of the South Asian MYBPC3Δ25bp Intronic Deletion in Hypertrophic Cardiomyopathy. Circ Genom Precis Med. 2020; 13. 10.1161/CIRCGEN.119.002783. 10.1161/CIRCGEN.119.002783 PMC7299222 32163302

[30] Morales J, Pujar S, Loveland JE, Astashyn A, Bennett R, Berry A (2022). A joint NCBI and EMBL-EBI transcript set for clinical genomics and research. Nature.

[31] Richards S, Aziz N, Bale S, Bick D, Das S, Gastier-Foster J (2015). Standards and guidelines for the interpretation of sequence variants: a joint consensus recommendation of the American College of Medical Genetics and Genomics and the Association for Molecular Pathology. Genet Med.

[32] Kelly MA, Caleshu C, Morales A, Buchan J, Wolf Z, Harrison SM (2018). Adaptation and validation of the ACMG/AMP variant classification framework for MYH7-associated inherited cardiomyopathies: recommendations by ClinGen’s Inherited Cardiomyopathy Expert Panel. Genet Med.

[33] Abou Tayoun AN, Pesaran T, DiStefano MT, Oza A, Rehm HL, Biesecker LG (2018). Recommendations for interpreting the loss of function PVS1 ACMG/AMP variant criterion. Hum Mutat.

[34] Brnich SE, Abou Tayoun AN, Couch FJ, Cutting GR, Greenblatt MS, Heinen CD, et al. Recommendations for application of the functional evidence PS3/BS3 criterion using the ACMG/AMP sequence variant interpretation framework. Genome Med. 2019; 12. 10.1186/S13073-019-0690-2. 10.1186/s13073-019-0690-2 PMC6938631 31892348

[35] Campuzano O, Sarquella-Brugada G, Fernandez-Falgueras A, Coll M, Iglesias A, Ferrer-Costa C, et al. Reanalysis and reclassification of rare genetic variants associated with inherited arrhythmogenic syndromes. EBioMed. 2020; 54. 10.1016/J.EBIOM.2020.102732. 10.1016/j.ebiom.2020.102732 PMC7136601 32268277

[36] Bournazos AM, Riley LG, Bommireddipalli S, Ades L, Akesson LS, Al-Shinnag M (2022). Standardized practices for RNA diagnostics using clinically accessible specimens reclassifies 75% of putative splicing variants. Genet Med.

[37] Lowey S, Bretton V, Gulick J, Robbins J, Trybus KM (2013). Transgenic mouse α- and β-cardiac myosins containing the R403Q mutation show isoform-dependent transient kinetic differences. J Biol Chem.

[38] Robinson P, Griffiths PJ, Watkins H, Redwood CS (2007). Dilated and hypertrophic cardiomyopathy mutations in troponin and alpha-tropomyosin have opposing effects on the calcium affinity of cardiac thin filaments. Circ Res.

[39] Karczewski KJ, Francioli LC, Tiao G, Cummings BB, Alföldi J, Wang Q (2020). The mutational constraint spectrum quantified from variation in 141,456 humans. Nature.

[40] Crotti L, Spazzolini C, Tester DJ, Ghidoni A, Baruteau AE, Beckmann BM (2019). Calmodulin mutations and life-threatening cardiac arrhythmias: insights from the International Calmodulinopathy Registry. Eur Heart J.

[41] Winbo A, Stattin EL, Nordin C, Diamant UB, Persson J, Jensen SM, et al. Phenotype, origin and estimated prevalence of a common long QT syndrome mutation: a clinical, genealogical and molecular genetics study including Swedish R518X/KCNQ1 families. BMC Cardiovasc Disord. 2014; 14. 10.1186/1471-2261-14-22. 10.1186/1471-2261-14-22 PMC3942207 24552659

[42] Ng K, Titus EW, Lieve KV, Roston TM, Mazzanti A, Deiter FH (2020). An international multicenter evaluation of inheritance patterns, arrhythmic risks, and underlying mechanisms of CASQ2-catecholaminergic polymorphic ventricular tachycardia. Circulation.

[43] Osborn DPS, Emrahi L, Clayton J, Tabrizi MT, Wan AYB, Maroofian R (2021). Autosomal recessive cardiomyopathy and sudden cardiac death associated with variants in MYL3. Genet Med.

[44] Jarvik GP, Browning BL (2016). Consideration of cosegregation in the pathogenicity classification of genomic variants. Am J Hum Genet.

[45] Jongbloed RJ, Marcelis CL, Doevendans PA, Schmeitz-Mulkens JM, Van Dockum WG, Geraedts JP (2003). Variable clinical manifestation of a novel missense mutation in the alpha-tropomyosin (TPM1) gene in familial hypertrophic cardiomyopathy. J Am Coll Cardiol.

[46] Shimamoto K, Ohno S, Kato K, Takayama K, Sonoda K, Fukuyama M (2022). Impact of cascade screening for catecholaminergic polymorphic ventricular tachycardia type 1 Arrhythmias and sudden death. Heart.

[47] Bagnall RD, Singer ES, Wacker J, Nowak N, Ingles J, King I, et al. Genetic Basis of Childhood Cardiomyopathy. Circ Genom Precis Med. 2022. 10.1161/CIRCGEN.121.003686. 10.1161/CIRCGEN.121.003686 36252119

[48] Deans ZC, Ahn JW, Carreira IM, Dequeker E, Henderson M, Lovrecic L (2022). Recommendations for reporting results of diagnostic genomic testing. Eur J Hum Genet.

[49] Cresswell L, Wallis Y, Fews G, Deans Z, Fratter C, Monkman L, et al. General Genetic Laboratory Reporting Recommendations(v1.1). 2020 https://www.acgs.uk.com/media/11649/acgs-general-genetic-laboratory-reporting-recommendations-2020-v1-1.pdf (accessed 28 Mar2022).

[50] Germain DP, Altarescu G, Barriales-Villa R, Mignani R, Pawlaczyk K, Pieruzzi F (2022). An expert consensus on practical clinical recommendations and guidance for patients with classic Fabry disease. Mol Genet Metab.

