# Supplementary material for: EMQN: Recommendations for genetic testing in inherited cardiomyopathies and arrhythmias
Source: Eur J Hum Genet. 2023 Jul 13;31(9):1003–9. doi: 10.1038/s41431-023-01421-w (PMC10474043; doi:10.1038/s41431-023-01421-w)
Supplement: Supplementary file 1 — Supplementary Appendices [file 41431_2023_1421_MOESM1_ESM.docx]

| **Appendix 1: Hypertrophic cardiomyopathy (HCM)** |
| --- |
| **Clinical summary** |
| **Key clinical features:** Left ventricular hypertrophy (LVH) in the absence of loading conditions, ≥1.5cm; myocyte disarray.  **Variable expressivity:** Signs and symptoms can be variable, even in relatives with the same pathogenic variant.  **Age-related and incomplete penetrance:** Usually develops after puberty but can appear at any age. Not all individuals with a pathogenic variant develop signs or symptoms, even at advanced age.  **Disease prevalence estimate/s:** ~1 in 500 to 1 in 200 [PMID:7641357; PMID: 25814232; PMID: 32895535]  **Note:** Estimates based on phenotype and genotype data from population-based cohorts.  **Clinical and genetic testing guidelines:**  **2022** EHRA/HRS/APHRS/LAHRS Expert consensus statement on the State of Genetic Testing for Cardiac Disease [PMID:3590533].  **2022** Interpretation and actionability of genetic variants in cardiomyopathies: a position statement from the European Society of Cardiology Council on cardiovascular genomics [PMID: 35089333].  **2020** AHA/ACC Guideline for the Diagnosis and Treatment of Patients with Hypertrophic Cardiomyopathy: A Report of the American College of Cardiology/American Heart Association Joint Committee on Clinical Practice Guidelines [PMID: 33229116].  **2019** Evaluating the Clinical Validity of Hypertrophic Cardiomyopathy Genes [PMID: 30681346].  **2014** ESC Guidelines on diagnosis and management of hypertrophic cardiomyopathy: the Task Force for the Diagnosis and Management of Hypertrophic Cardiomyopathy of the European Society of Cardiology (ESC) [PMID: 25173338]. |
| **Genetic summary** |
| **Genetic basis:** Predominantly caused by pathogenic variants in genes encoding protein components of the cardiac sarcomere. Pathogenic variants in non-sarcomeric genes have also been detected in individuals referred for HCM genetic testing (see **Table 2. Differential diagnoses**.)  **Key genes and mode of inheritance:** Please refer to **Table 1.**  **Genotype-phenotype:** For the key sarcomeric genes, there are no robust genotype-phenotype relationships that could reliably be used to inform test strategy or aid results interpretation.  **Disease-specific variant interpretation guidelines:**  **2017** Adaptation and validation of the ACMG/AMP variant classification framework for MYH7-associated inherited cardiomyopathies: recommendations by ClinGen's Inherited Cardiomyopathy Expert Panel. [PMID: 29300372] |
| **Differential diagnoses** |
| Other genetic disorders can present with apparently isolated left ventricular hypertrophy similar to that observed in individuals with sarcomeric HCM (see **Table 2. Differential diagnosis**). In individuals referred for HCM genetic testing from specialist centres, the estimated diagnostic yield in these genes is expected to be very low; however, testing could provide clinically important diagnosis and change patient management. As such, these genes may be considered for inclusion in HCM test panels. |
| **Please note:** *This disease specific summary is provided as an appendix to the main best practice guideline. Users should refer to the main document for recommendations on analysis, interpretation and reporting in inherited cardiomyopathies and arrhythmias.*  *The information in this appendix is based on current evidence and existing clinical and variant interpretation guidelines.*  *The core gene list (****Table 1****) takes into consideration existing ClinGen gene-disease clinical validity curations and may change as data from ongoing curation efforts becomes available.* |

**Disease specific summary**: Hypertrophic cardiomyopathy **Version:** March 2022. Page **1** of **3**

| **Gene** | **Transcripts** | | **Predominant type of pathogenic variant** | **Inheritance** | **Diagnostic Yield** |
| --- | --- | --- | --- | --- | --- |
| *ACTC1* | ENST00000290378.6 | NM_005159.5 | Non-truncating | AD | 0.1-1% |
| *MYBPC3* | ENST00000545968.6;  ENST00000545968.6: c.1224-52G>A**^a^** | NM_000256.3;  NM_000256.3: c.1224-52G>A^a^ | Truncating; Non-truncating | AD | 15-20% |
| *MYH7* | ENST00000355349.4 | NM_000257.4 | Non-truncating | AD | 10-15% |
| *MYL2* | ENST00000228841.15 | NM_000432.4 | Non-truncating | AD; AR | <0.1% |
| *MYL3* | ENST00000292327.6 | NM_000258.3 | Non-truncating | AD; AR | <0.1% |
| *TNNI3* | ENST00000344887.10 | NM_000363.5 | Non-truncating | AD | 1-2% |
| *TNNT2* | ENST00000656932.1 | NM_001276345.2 | Non-truncating | AD | 1-2% |
| *TPM1* | ENST00000403994.9 | NM_001018005.2 | Non-truncating | AD | 0.1-1% |
| **Table 1.** Listed here are the ‘Definitive’ sarcomeric HCM genes recommended for inclusion in HCM test panels [PMID: 30681346]. Other definitive syndromic and intrinsic cardiomyopathy genes may also be considered for HCM test panels (see Table 2: Differential Diagnoses). **Transcripts:** Preferred transcripts for defining the regions of interest (ROI) for variant calling, and for variant annotation. Listed are the Ensembl (<https://www.ensembl.org>) and RefSeq (<https://www.ncbi.nlm.nih.gov/refseq/>) transcripts specified by the MANE collaboration (v0.95) [PMID: 35388217] and transcripts containing coding regions not included in the MANE transcripts (e.g. tissue-specific exons, developmental stage-specific exons) but considered relevant to clinical testing in the context of cardiac disease. In most clinical laboratories, the ROI includes the coding regions and adjacent intronic sequence (e.g.  ± 10 to 20 nucleotides) of these transcripts. **Predominant type of pathogenic variant:** 'Truncating' includes out-of-frame insertions/deletions; variants leading to aberrant splicing (canonical splice sites or other 'deep' intronic variant); nonsense variants; copy number variants (CNVs) involving deletion of multiple exons within a gene or a whole gene. 'Non-truncating' includes missense variants and small in-frame insertions/deletions.  **Inheritance:** AD=autosomal dominant; AR = autosomal recessive. **Diagnostic yield:**  *Approximate* proportion of probands referred for HCM testing in whom ‘likely pathogenic’, or ‘pathogenic variants’ are detected.  PMID =PubMed ID <https://pubmed.ncbi.nlm.nih.gov/>. **Note^a^:** The “c.” coordinates of the common pathogenic *MYBPC3* variant, c.1224-52G>A, detected in ~1% of individuals referred for HCM testing [PMID:32163302; PMID: 32396390], which lies in flanking intronic region outside standard ROIs, has also been included. *Please note:* less frequent pathogenic variants have been detected in other intronic regions of *MYBPC3,* in particular in the intronic regions flanking the micro-exons (exons 10, 11, 14 and 20); therefore, test sensitivity may be increased by extending the ROI in these regions (e.g.  ± 100 nucleotides). | | | | | |

**Disease specific summary**: Hypertrophic cardiomyopathy **Version:** March 2022. Page **2** of **3**

| **Disease** | **Gene** | **Gene OMIM** | **Diagnostic yield** | **Mode of inheritance** |
| --- | --- | --- | --- | --- |
| Fabry disease | *GLA* | #300644 | 0.1-0.5% | XL |
| *FHL1*-related muscular dystrophy | *FHL1* | #300163 | <0.1% | XL |
| *FLNC*-related filaminopathy | *FLNC* | #102565 | <0.1% | AD |
| Danon disease | *LAMP2* | #309060 | <0.1% | XL |
| *PLN-*related cardiomyopathy | *PLN* | #172405 | <0.1% | AD |
| *PRKAG2-*related cardiomyopathy | *PRKAG2* | #602743 | <0.1% | AD |
| Noonan syndrome | *PTPN11* | #176876 | <0.1% | AD |
| Noonan syndrome | *RAF1* | #164760 | <0.1% | AD |
| Noonan syndrome | *RIT1* | #609591 | <0.1% | AD |
| Transthyretin amyloidosis | *TTR* | #176300 | <0.1% | AD |

**Table 2. Differential diagnosis.**Listed here are the ClinGen ‘Definitive’ intrinsic cardiomyopathy or syndromic genes most frequently included in HCM gene panels [PMID:30681346]. **Gene OMIM:** Online Mendelian Inheritance in Man (<https://omim.org>). **Diagnostic yield:** *Approximate* proportion of probands referred for HCM testing in whom ‘likely pathogenic’, or ‘pathogenic variants’ are detected.  **Inheritance:** AD=autosomal dominant; AR = autosomal recessive; *XL=* X-linked*.* Referral criteria for HCM testing can differ between centres, and not all these genes are included in HCM test panels, therefore estimates of diagnostic yield can vary. PMID =PubMed ID <https://pubmed.ncbi.nlm.nih.gov/>

| **Appendix 2: Dilated cardiomyopathy (DCM)** |
| --- |
| **Clinical summary** |
| **Key clinical features:** Ventricular dilation and systolic dysfunction (conventionally, a left-ventricular ejection fraction of <50%), idiopathic or in the absence of other causal factors.  **Variable expressivity:** Signs and symptoms can be variable, even in relatives with the same pathogenic variant.  **Age-related and incomplete penetrance:** Usually develops after puberty but can appear at any age. Not all individuals with a pathogenic variant develop signs or symptoms, even at advanced age.  **Disease prevalence estimate/s:** ~1 in 500 to 1 in 250 [PMID: 23900355; PMID: 32895535].  **Clinical and genetic testing guidelines:**  **2022** EHRA/HRS/APHRS/LAHRS Expert consensus statement on the State of Genetic Testing for Cardiac Disease [PMID:3590533].  **2022** Interpretation and actionability of genetic variants in cardiomyopathies: a position statement from the European Society of Cardiology Council on cardiovascular genomics [PMID: 35089333].  **2021** Evidence-Based Assessment of Genes in Dilated Cardiomyopathy [PMID:33947203].  **2019** HRS expert consensus statement on evaluation, risk stratification, and management of arrhythmogenic cardiomyopathy [PMID:31078652]. |
| **Genetic summary** |
| **Genetic basis:** Caused by pathogenic variants in genes encoding proteins involved in a diverse range of cardiac muscle cell functions (e.g., sarcomeric, nuclear envelope, Z-disc, cytoskeleton, desmosome, co-chaperone, RNA-binding, junctional and genes, ion channel).  **Key genes and mode of inheritance:** Please refer to **Table 1.**  **Genotype-phenotype:** Gene-specific characteristics, particularly relating to arrhythmic phenotypes, have been described (see **Table 2**); however, generally, this information is not used to direct genetic testing strategy.    **Disease-specific variant interpretation guidelines:**  **2017** Adaptation and validation of the ACMG/AMP variant classification framework for MYH7-associated inherited cardiomyopathies: recommendations by ClinGen's Inherited Cardiomyopathy Expert Panel [PMID: 29300372].  **2020** Variant Interpretation for Dilated Cardiomyopathy: Refinement of the American College of Medical Genetics and Genomics/ClinGen Guidelines for the DCM Precision Medicine Study [PMID: 32160020]. |
| **Differential diagnoses** |
| DCM is a highly heterogeneous condition with both genetic and non-genetic causes. Due to overlapping clinical features, hypertrophic cardiomyopathy (HCM) and arrhythmogenic right ventricular cardiomyopathy (ARVC) genes may be included in DCM panels. DCM is also a feature of several rare, multi-system, syndromic disorders and can be the primary presentation. Genetic testing beyond idiopathic isolated DCM genes may be relevant in some cases and should be guided by clinical and family history. |
| ***Please note:*** *This disease-specific summary is provided as an appendix to the main best practice guideline. Users should refer to the main document for recommendations on analysis, interpretation and reporting in inherited cardiomyopathies and arrhythmias.*  *The information in this appendix is based on current evidence and existing clinical and variant interpretation guidelines.*  *The core gene list (****Table 1****) takes into consideration existing ClinGen gene-disease clinical validity curations and may change as data from ongoing curation efforts becomes available.* |

| **Gene** | **Transcripts** | | **Predominant type of pathogenic variant** | **Inheritance** | **Diagnostic Yield** |
| --- | --- | --- | --- | --- | --- |
| **Definitive/Strong evidence genes** | | | | | |
| *BAG3* | ENST00000369085.8 | NM_004281.4 | Truncating | AD | 2-4% |
| *DES* | ENST00000373960.4 | NM_001927.4 | Non-truncating | AD | 2-4% |
| *DSP* | ENST00000379802.8 | NM_004415.4 | Truncating | AD | unknown |
| *FLNC* | ENST00000325888.13**^a^** | NM_001458.5**^a^** | Truncating | AD | 2-4% |
| *LMNA* | ENST00000368300.9  ENST00000677389.1 | NM_170707.4  NM_005572.4 | Truncating & Non-truncating | AD | 6% |
| *MYH7* | ENST00000355349.4 | NM_000257.4 | Non-truncating | AD | 5-6% |
| *PLN* | ENST00000357525.6 | NM_002667.5 | Non-truncating | AD | <0.5%**^g^** |
| *RBM20* | ENST00000369519.4 | NM_001134363.3 | Non-truncating | AD | 1-2% |
| *SCN5A* | ENST00000423572.7**^b^**  ENST00000413689.6**^c^** | NM_00335.5 **^b^**  NM_001099404.2**^c^** | Non-truncating | AD | 2-3% |
| *TNNC1* | ENST00000232975.8 | NM_003280.3 | Non-truncating | AD | <1% |
| *TNNT2* | ENST00000656932.1 | NM_001276345.2 | Non-truncating | AD | 2-4% |
| *TTN* | ENST00000591111.5 **^d^**  ENST00000589042.5**^e^** | NM_001256850.1 **^d^**  NM_001267550.2**^e^** | Truncating**^f^** | AD | 15-20% |
| **Moderate evidence genes** | | | | | |
| *ACTC1* | ENST00000290378.6 | NM_005159.5 | Non-truncating | AD | <1% |
| *ACTN2* | ENST00000366578.6 | NM_001103.4 | Non-truncating & Truncating | AD | unknown |
| *JPH2* | ENST00000372980.4 | NM_020433.5 | Non-truncating & Truncating | AD | unknown |
| *NEXN* | ENST00000334785.12 | NM_144573.4 | Non-truncating & Truncating | AD | unknown |
| *TNNI3* | ENST00000344887.10 | NM_000363.5 | Non-truncating | AD | <1% |
| *TPM1* | ENST00000403994.9 | NM_001018005.2 | Non-truncating | AD | <1% |
| *VCL* | ENST00000211998.10 | NM_014000.3 | Truncating | AD | unknown |
| **Other** | | | | | |
| *DMD* | ENST00000357033.9 | NM_004006.3 | Truncating; Non-truncating | XL | <0.5% |
| **Table 1.** Listed here are the ‘Definitive / Strong’ and ‘Moderate’ evidence genes recommended for inclusion in idiopathic, isolated DCM [PMID:33947203]. The *DMD* gene was not reviewed in PMID:33947203 but has been included here on the basis of existing evidence that variants in this gene are causal of X-linked isolated DCM [PMID:21851881; PMID: 27761893; PMID: 34050592]. **Transcripts:** Preferred transcripts for defining the regions of interest (ROI) for variant calling, and for variant annotation. Listed are Ensembl (<https://www.ensembl.org>) and RefSeq (<https://www.ncbi.nlm.nih.gov/refseq/>) transcripts specified by the MANE collaboration (v0.95) [PMID: 35388217] and transcripts containing coding regions not included in the MANE transcripts (e.g. tissue-specific exons, developmental stage-specific exons) but considered relevant to clinical testing in the context of cardiac disease. In most clinical laboratories, the ROI includes the coding regions and adjacent intronic sequence (e.g.  ± 10 to 20 nucleotides) of these transcripts. **Predominant type of pathogenic variant:** 'Truncating' includes out-of-frame insertions/deletions; variants leading to aberrant splicing (canonical splice sites or other 'deep' intronic variant); nonsense variants; copy number variants (CNVs) involving deletion of multiple exons within a gene or a whole gene. 'Non-truncating' includes missense variants and small in-frame insertions/deletions. **Inheritance:** AD=autosomal dominant; AR = autosomal recessive., XL = X-linked. **Diagnostic yield:**  *Approximate* proportion of probands referred for DCM testing in whom ‘likely pathogenic’, or ‘pathogenic variants’ are detected. **Note^a^**: *FLNC* exon 46 (GRCh37:g.128,497,172 to g.128,497,390; GRCh38:g.128,857,118 to g.128,857,336), exon 47 (GRCh37:g.128,498,062 to g.128,498,271; GRCh38:g.128,858,008 to g.128,858,217) and exon 48 (GRCh37:g.128,498,390 to g.128,498,577; GRCh38:g.128,858,336 to g.128,858,523) show high (98%) homology to a downstream pseudogene region: care should be taken when assessing variants in these regions [PMID: 20578970]. **Note^b^**: *SCN5A* adult isoform, exon 6. **Note^c^:** *SCN5A* fetal isoform, with alternative exon 6 (denoted exon 6a). **Note^d^**: The ENST00000591111.5/NM_001256850.1 transcripts, which include the exons in the two principal adult cardiac isoforms (N2BA and N2B) are considered more appropriate for variant calling and interpretation in the context of DCM. **Note^e^**: The MANE Select transcripts for *TTN* (ENST00000589042.5/ NM_001267550.2) represent a manually curated inferred ‘meta-transcript’, incorporating all exons of all known isoforms (including fetal and non-cardiac isoforms); some of the exons in these transcripts are not relevant in the context of DCM and therefore clinical laboratories may prefer not to use these transcripts to define the ROI for variant calling. The MANE Plus Clinical transcripts (ENST00000360870.10/NM_133379.5), which represent the NOVEX3 isoform, are not routinely used for clinical genetic testing in the context of DCM due to lack of evidence for pathogenicity of truncating variants in regions unique to this isoform. **Note^f^**: Currently, in the context of DCM, the strongest evidence of pathogenicity is for variants located in exons encoding the N2B unique sequence, the distal I-band, and the A-band domains of the titin protein [PMID:25589632; PMID:27869827; PMID:29238064]. **Note^g^:** Founder variant described with higher frequency in the Netherlands (*PLN* p.(Arg14del) [PMID:23568436]. PMID =PubMed ID <https://pubmed.ncbi.nlm.nih.gov/> | | | | | |

| **Gene** | **Genotype-phenotype characteristics** |
| --- | --- |
| *DES* | Arrhythmic phenotype.  Cardiac conduction-system abnormalities.  Skeletal myopathy.  [PMID:33373648; PMID:29274115] |
| *DSP* | Arrhythmic phenotype. High incidence of ventricular arrhythmias.  Arrhythmogenic right ventricular cardiomyopathy (ARVC).  Autosomal dominant and recessive cardiocutaneous syndromes.  [PMID: 3237266; OMIM:125647] |
| *FLNC* | Arrhythmic phenotype. High incidence of ventricular arrhythmias.  [PMID:27908349; PMID:30067491] |
| *LMNA* | Arrhythmic phenotype. Cardiac conduction-system abnormalities. High incidence of SCD.  Muscular dystrophy.  Lipodystrophy.  [PMID:20301717] |
| *PLN* | Arrhythmic phenotype.  [PMID:30547415] |
| *RBM20* | Arrhythmic phenotype. High incidence of SCD.  [PMID:30871351] |
| *SCN5A* | Arrhythmic phenotype.  Cardiac conduction-system abnormalities.  Ventricular premature beats.  [PMID:34949099]. |
| *TTN* | Arrhythmic phenotype.  [PMID:31251381] |

**Table 2. Genotype-phenotype characteristics described in key DCM genes.** SCD=sudden cardiac death.

| **Appendix 3: Arrhythmogenic right ventricular cardiomyopathy (ARVC)** |
| --- |
| **Clinical summary** |
| **Key clinical features:** Ventricular tachycardia, t-wave inversion, progressive myocardial fibro-fatty replacement in the right ventricular (RV) myocardium.  **Variable expressivity:** Signs and symptoms can be variable, even in relatives with the same pathogenic varian**t.**  **Age-related and incomplete penetrance:** Typically manifests in the second to fifth decade. Not all individuals with a pathogenic variant develop signs or symptoms, even at advanced age.  **Disease prevalence estimate/s:** ~1 in 5,000 to 1 in 1,000 [PMID: 28912183; PMID: 32895535].  **Note:** Higher prevalence in some populations due to founder variants (Naxos, Greece; Newfoundland, North America; Netherlands).  **Clinical and genetic testing guidelines:**  **2022** EHRA/HRS/APHRS/LAHRS Expert consensus statement on the State of Genetic Testing for Cardiac Disease [PMID:3590533].  **2022** Interpretation and actionability of genetic variants in cardiomyopathies: a position statement from the European Society of Cardiology Council on cardiovascular genomics [PMID: 35089333].  **2021** International Evidence Based Reappraisal of Genes Associated with Arrhythmogenic Right Ventricular Cardiomyopathy Using the Clinical Genome Resource Framework [PMID: 33831308].  **2020** Arrhythmogenic right ventricular cardiomyopathy: evaluation of the current diagnostic criteria and differential diagnosis [PMID: 31637441].  **2019** HRS expert consensus statement on evaluation, risk stratification, and management of arrhythmogenic cardiomyopathy [PMID:31078652].  **2010** Diagnosis of arrhythmogenic right ventricular cardiomyopathy/dysplasia: proposed modification of the task force Criteria [PMID: 20172912; PMID:20172911]. |
| **Genetic summary** |
| **Genetic basis:** Predominantly caused by pathogenic variants in genes encoding protein components of the cardiac desmosome (e.g., *PKP2*, *DSP*, *DSG2*, *DSC2*, *JUP*). Pathogenic variants in non-desmosomal genes (e.g., *TMEM43*, *PLN*, *FLNC, DES*) are less frequently detected in individuals referred for ARVC genetic testing, although some founder variants may be frequently detected in specific populations (e.g., *TMEM43*, *PLN*).  **Key genes and mode of inheritance:** Please refer to **Table 1.**  **Genotype-phenotype:**  Gene-specific correlations have been described; however, generally, this information is not used to direct genetic testing strategy. In ‘classic’ desmosomal ARVC the right ventricle is predominantly affected; however, biventricular or left-dominant forms are also described. The presence of cutaneous features, such as woolly hair and palmoplantar keratoderma, in addition to cardiomyopathy, are consistent with autosomal recessive cardiocutaneous syndromes (see **Table 2**).  **Disease-specific variant interpretation guidelines:** None currently available. |
| **Differential diagnoses** |
| Other genetic disorders can present with arrhythmogenic cardiomyopathy similar to that observed in individuals with ‘classic’ desmosomal ARVC (e.g., cardiac disorders such as dilated cardiomyopathy and Brugada syndrome, or neuromuscular disorders such as filaminopathy and laminopathy). Given this clinical and genetic heterogeneity, genetic testing of additional dilated and arrhythmogenic cardiomyopathy genes may be relevant in some cases. |
| **Please note:** *This disease-specific summary is provided as an appendix to the main best practice guideline. Users should refer to the main document for recommendations on analysis, interpretation and reporting in inherited cardiomyopathies and arrhythmias.*  *The information in this appendix is based on current evidence and existing clinical and variant interpretation guidelines.*  *The core gene list (****Table 1****) takes into consideration existing ClinGen gene-disease clinical validity curations and may change as data from ongoing curation efforts becomes available.* |

| **Gene** | **Transcripts** | | | | **Predominant type of pathogenic variant** | **Inheritance** | **Diagnostic Yield** |
| --- | --- | --- | --- | --- | --- | --- | --- |
| **Definitive evidence genes** | | | | | | | |
| *PKP2* | ENST000000340811.9^a^  ENST0000070846.11^b^ | | | NM_001005242.3 **^a^**  NM_004572.3**^b^** | Truncating | AD | 10-45% |
| *DSP* | ENST00000379802.8 | | | NM_004415.4 | Truncating; Non-truncating | AD; AR | 10-15% |
| *DSG2* | ENST00000261590.13 | | | NM_001943.5 | Truncating; Non-truncating | AD; AR | 7-10% |
| *DSC2* | ENST00000251081[isoform-b]**^c^**  ENST00000280904.11 [isoform-a] | | | NM_004949[isoform-b] **^c^**  NM_024422.6[isoform-a] | Truncating; Non-truncating | AD; AR | 2% |
| *JUP* | ENST00000393931.8 | | | NM_002230.4 | Truncating; Non-truncating | AR | <0.1%**^e^** |
| *TMEM43* | ENST00000306077.5 | | | NM_024334.3 | Non-truncating | AD | <0.1%**^e^** |
| **Moderate evidence genes** | | | | | | | |
| *PLN* | ENST00000357525.6 | | | NM_002667.5 | Non-truncating | AD | <0.1%**^e^** |
| *DES* | ENST00000373960.4 | | | NM_001927.4 | Non-truncating | AD | <0.1% |
| **Other** | | | | | | | |
| *FLNC* | ENST00000325888.13^d^ | | | NM_001458.5**^d^** | Truncating | AD | 1% |
| **Table 1** Listed here are the ‘Definitive’ (*PKP2*, *DSP*, *DSG2*, *DSC2*, *JUP,* *TMEM43*) and ‘Moderate’ (*PLN*, *DES*) evidence genes recommended for inclusion in ARVC test panels by the Clinical Genome Resource (ClinGen) expert panel [PMID: 33831308]. The *FLNC* gene was not reviewed in PMID:33831308 but has been included here on the basis of existing evidence that variants in this gene are causal of arrhythmogenic cardiomyopathy and have been detected in individuals referred for ARVC genetic testing [PMID: 27908349; PMID: 30067491; PMID:31924696]. **Transcripts:** Preferred transcripts for defining the regions of interest (ROI) for variant calling, and for variant annotation. Listed are the Ensembl (<https://www.ensembl.org>) and RefSeq (<https://www.ncbi.nlm.nih.gov/refseq/>) transcripts specified by the MANE collaboration (v0.95) [PMID: 35388217] and transcripts containing coding regions not included in the MANE transcripts (e.g. tissue-specific exons, developmental stage-specific exons) but considered relevant to clinical testing in the context of cardiac disease. In most clinical laboratories, the ROI includes the coding regions and adjacent intronic sequence (e.g.  ± 10 to 20 nucleotides) of these transcripts. **Predominant type of pathogenic variant:** 'Truncating' includes out-of-frame insertions/deletions; variants leading to aberrant splicing (canonical splice sites or other 'deep' intronic variant); nonsense variants; copy number variants (CNVs) involving deletion of multiple exons within a gene or a whole gene. 'Non-truncating' includes missense variants and small in-frame insertions/deletions.  **Inheritance:** AD=autosomal dominant; AR = autosomal recessive; *de novo*=high proportion of de novo variants. **Diagnostic yield:**  *Approximate* proportion of probands referred for ARVC testing in whom ‘likely pathogenic’, or ‘pathogenic variants’ are detected. **Note^a^**: MANE transcripts should be used to define the ROI for variant calling in this gene. **Note^b^:** Transcripts include a region that has low expression in cardiac tissue and is not considered relevant to clinical testing (GRCh37:g.32,996,116 to g.32,996,247; GRCh38: g.32,843,182 to g.32,843,313) [PMID:21378009]; to maintain consistency with the existing literature, these transcripts may be preferred for variant annotation. **Note^c^:** There are two isoforms of *DSC2* (DSC2a and DSC2b) which differ at their C-terminal regions. The longer DCS2a isoform has been selected as the MANE transcript, however, the shorter DSC2b isoform shows higher expression in the heart; therefore, the b transcript is relevant for clinical testing [PMID:20197793]. **Note^d^:** *FLNC* exon 46 (GRCh37:g.128,497,172 to g.128,497,390; GRCh38:g.128,857,118 to g.128,857,336), exon 47 (GRCh37:g.128,498,062 to g.128,498,271; GRCh38:g.128,858,008 to g.128,858,217) and exon 48 (GRCh37:g.128,498,390 to g.128,498,577; GRCh38:g.128,858,336 to g.128,858,523) show high (98%) homology to a downstream pseudogene region: care should be taken when assessing variants in these regions [PMID: 20578970]. **Note^e^:** Founder variants described with higher frequency in specific populations e.g. *JUP* p.(Glu301Gly) (French-Canadian, PMID:28098346); *TMEM43* p.(Ser358Leu) (Newfoundland, PMID:18313022); *PLN* p.(Arg14del) (Netherlands, PMID:23568436); PMID =PubMed ID <https://pubmed.ncbi.nlm.nih.gov/>. | | | | | | | |
| **Gene** | | **Predominant ventricle** | **Genotype-phenotype characteristics** | | | | |
| *PKP2* | | RV, BV | ‘Classic’ ARVC phenotype. | | | | |
| *DSP* | | LV, BV, RV | Arrhythmogenic right ventricular cardiomyopathy (ARVC).  Arrhythmogenic cardiomyopathy (ACM).  Dilated cardiomyopathy (DCM).  Autosomal dominant and recessive cardiocutaneous syndrome [OMIM:125647]. | | | | |
| *DSG2* | | RV, LV, BV | Autosomal dominant and recessive Arrhythmogenic right ventricular cardiomyopathy (ARVC) [PMID: 20031616]. | | | | |
| *DSC2* | | RV, BV | Autosomal dominant and recessive Arrhythmogenic right ventricular cardiomyopathy (ARVC) [PMID: 20031616].  Autosomal recessive cardiocutaneous syndrome [PMID: 18957847]. | | | | |
| *JUP* | | RV, BV | Autosomal recessive Naxos Disease (cardiocutaneous syndrome characterised by ARVC, palmoplantar keratoderma and woolly hair) [PMID:34776086]. | | | | |
| *TMEM43* | | RV, BV | High incidence of SCD [PMID:18313022]. | | | | |
| *PLN* | | LV, BV | Arrhythmogenic right ventricular cardiomyopathy (ARVC).  Arrhythmogenic cardiomyopathy (ACM).  Dilated cardiomyopathy (DCM).  [PMID:30547415] | | | | |
| *DES* | | LV, BV | Arrhythmogenic right ventricular cardiomyopathy (ARVC).  Arrhythmogenic cardiomyopathy (ACM).  Dilated cardiomyopathy (DCM).  Cardiac conduction-system abnormalities.  Skeletal myopathy.  [PMID:33373648; PMID:29274115] | | | | |
| *FLNC* | | LV | Arrhythmogenic right ventricular cardiomyopathy (ARVC).  Arrhythmogenic cardiomyopathy (ACM).  Dilated cardiomyopathy (DCM).  [PMID:27908349; PMID:30067491] | | | | |

**Table 2. Genotype-phenotype characteristics described in core ARVC genes** [PMID: 34065276, PMID:34352074]. RV=Right ventricle, LV=Left ventricle, BV=Bi-ventricular.

SCD=sudden cardiac death.

| **Appendix 4: Long QT syndrome (LQTS)** |
| --- |
| **Clinical summary** |
| **Key clinical features:** Prolonged QT interval and polymorphic ventricular arrhythmia (torsade de pointes) on ECG. Recurrent syncope, seizure, sudden death.  **Variable expressivity:** Signs and symptoms can be variable, even in relatives with the same pathogenic variant.  **Age-related and incomplete penetrance:** Typically manifests in childhood and adolescence but can appear at any age. Not all individuals with a pathogenic variant develop signs or symptoms, even at advanced age. Recessive forms can appear in infancy.  **Disease prevalence estimate/s:** ~1 in 3,000 to 1/2,000 [PMID:22895603].  **Clinical and genetic testing guidelines:**  **2022** EHRA/HRS/APHRS/LAHRS Expert consensus statement on the State of Genetic Testing for Cardiac Disease [PMID:3590533].  **2022** Interpretation and actionability of genetic variants in cardiomyopathies: a position statement from the European Society of Cardiology Council on cardiovascular genomics [PMID: 35089333].  **2020** An International, Multicentered, Evidence-Based Reappraisal of Genes Reported to Cause Congenital Long QT Syndrome [PMID: 31983240].  **2013** HRS/EHRA/APHRS Expert Consensus Statement on the Diagnosis and Management of Patients with Inherited Primary Arrhythmia Syndromes [PMID: 24011539]. |
| **Genetic summary** |
| **Genetic basis:** Predominantly caused by pathogenic variants in genes encoding protein components of cardiac potassium, sodium and calcium ion channels, or proteins that interact with these ion channels.  **Key genes and mode of inheritance:** Please refer to **Table 1.**  **Genotype-phenotype:** Gene-specific correlations have been described (see **Table 2**); however, generally, this information is not used to direct genetic testing strategy.  **Disease-specific variant interpretation guidelines:** None currently available. |
| **Differential diagnoses** |
| Rare, atypical, syndromic, and acquired forms of LQTS have been described and these genes may be considered for inclusion in LQTS test panels (see **Table 3**). |
| **Please note:** *This disease -specific summary is provided as an appendix to the main best practice guideline. Users should refer to the main document for recommendations on analysis, interpretation and reporting in inherited cardiomyopathies and arrhythmias.*  *The information in this appendix is based on current evidence and existing clinical and variant interpretation guidelines.*  *The core gene list (****Table 1****) takes into consideration existing ClinGen gene-disease clinical validity curations and may change as data from ongoing curation efforts becomes available.* |

| **Gene** | **Transcripts** | | **Predominant type of pathogenic variant** | **Inheritance** | **Diagnostic Yield** |
| --- | --- | --- | --- | --- | --- |
| **Typical LQTS** | | | | | |
| *KCNH2* | ENST00000262186.10 | NM_000238.4 | Truncating; Non-truncating | AD | 25-30% |
| *KCNQ1* | ENST00000155840.12 | NM_000218.3 | Truncating; Non-truncating | AD; AR**^a^** | 30-35% |
| *SCN5A* | ENST00000423572.7**^b^**  ENST00000413689.6**^c^** | NM_00335.5 **^b^**  NM_001099404.2**^c^** | Non-truncating | AD | 5-10% |
| **Table 1.** Listed here are the ‘Definitive’ genes which should be included in test panels [PMID:31983240]. Other ‘Definitive’ genes which cause rare atypical and syndromic forms of LQTS may also be considered for inclusion in LQTS test panels (see Table 3). **Transcripts:** Preferred transcripts for defining the regions of interest (ROI) for variant calling, and for variant annotation. Listed are the Ensembl (<https://www.ensembl.org>) and RefSeq (<https://www.ncbi.nlm.nih.gov/refseq/>) transcripts specified by the MANE collaboration (v0.95) [PMID: 35388217]. In most clinical laboratories, the ROI includes the coding regions and adjacent intronic sequence (e.g.  ± 10 to 20 nucleotides) of these transcripts. **Predominant type of pathogenic variant:** 'Truncating' includes out-of-frame insertions/deletions; variants leading to aberrant splicing (canonical splice sites or other 'deep' intronic variant); nonsense variants; copy number variants (CNVs) involving deletion of multiple exons within a gene or a whole gene. 'Non-truncating' includes missense variants and small in-frame insertions/deletions. **Inheritance:** AD=autosomal dominant. AR=autosomal recessive. **Diagnostic yield:**  *Approximate* proportion of probands referred for LQTS testing in whom ‘likely pathogenic’, or ‘pathogenic variants’ are detected.  PMID =PubMed ID <https://pubmed.ncbi.nlm.nih.gov/>. **Note^a^:** rare homozygous and compound heterozygous cases described e.g., PMID:24400285; PMID:19027783, PMID:28944242. **Note^b^**: *SCN5A* adult isoform, exon 6. **Note^c^:** *SCN5A* fetal isoform, with alternative exon 6 (denoted exon 6a). | | | | | |

| **Gene** | **Triggers** | **T-wave morphology** |
| --- | --- | --- |
| *KCNQ1* | Physical and emotional stress, swimming | Broad-based T-waves. |
| *KCNH2* | Auditory stimuli (e.g., sudden noise). | Low-amplitude notched T-wave. |
| *SCN5A* | Sleep/rest. | Late-onset peaked/biphasic T-waves. |

**Table 2. Genotype-phenotype characteristics described in key Long QT syndrome (LQTS) genes** [PMID: 24093767]. Generally, this information is not used to direct genetic testing strategy.

| **Disease** | **Gene** | **Gene OMIM** | **Diagnostic yield** | **Mode of inheritance** |
| --- | --- | --- | --- | --- |
| **Acquired LQTS** | | | | |
| Acquired long QT syndrome | *KCNE1* | 176261 | <0.5% | AD  (Very low/incomplete penetrance) |
| Acquired long QT syndrome | *KCNE2* | 603796 | <0.5% | AD  (Very low/incomplete penetrance) |
| **Atypical LQTS** | | | | |
| LQTS presenting in infancy or early childhood with heart block and severe QT prolongation. | *CALM1*  *CALM2*  *CALM3* | 114180  114182  114183 | Unknown | AD  (Predominantly *de novo*) |
| QT prolongation, negative T waves in precordial leads and exercise-induced arrhythmia in early childhood.  Also detected in cases with catecholaminergic Polymorphic Ventricular Tachycardia (CPVT). | *TRDN* | 603283 | Unknown | AR |
| Variable cardiac arrhythmia phenotypes, including those typical of LQTS and CPVT. | *TECRL* | 617242 | Unknown | AR |
| **Syndromic LQTS** | | | | |
| Timothy syndrome | *CACNA1C* | 114205 | <0.01% | AD  (Predominantly *de novo*) |
| Andersen-Tawil syndrome | *KCNJ2* | 600681 | <0.01% | AD |
| Jervell & Lange-Nielson syndrome | *KCNQ1 KCNE1* | 607542  176261 | <0.01% | AR |

**Table 3. Rare, atypical and syndromic forms of LQTS.**Listed here are the ‘Definitive’ and ‘Strong’ genes associated with acquired, atypical and syndromic forms of Long QT syndrome (LQTS) [PMID: 31983240]. Note: Jervell and Lange-Nielson syndrome was not curated in this publication. OMIM: Online Mendelian Inheritance in Man (<https://omim.org>) **Diagnostic yield:** *Approximate* proportion of probands referred for LQTS testing in whom ‘likely pathogenic’, or ‘pathogenic variants’ are detected.  **Inheritance:** AD=autosomal dominant; AR = autosomal recessive. Referral criteria for LQTS testing can differ between centres, and not all these genes are routinely included in LQTS test panels, therefore estimates of diagnostic yield can vary. PMID =PubMed ID <https://pubmed.ncbi.nlm.nih.gov/>

| **Appendix 5: Brugada syndrome (BrS)** |
| --- |
| **Clinical summary** |
| **Key clinical features:** Type 1 ST-segment elevation (in precordial leads V1 to V3) on ECG (spontaneous or drug-induced). Ventricular fibrillation (VF) or aborted sudden death (more often nocturnal); syncope; palpitations; nocturnal agonal respiration.  **Variable expressivity:** Signs and symptoms can be variable, even in relatives with the same pathogenic variant.  **Age-related and incomplete penetrance:** Typically manifests in adulthood (3^rd^ to 4^th^ decade) but can appear at any age. Not all individuals with a pathogenic variant develop signs or symptoms, even at advanced age.  **Disease prevalence estimate/s:** ~1 in 5,000 to 1 in 2,000 [PMID:30139433].  **Clinical and genetic testing guidelines:**  **2022** EHRA/HRS/APHRS/LAHRS Expert consensus statement on the State of Genetic Testing for Cardiac Disease [PMID:3590533].  **2022** Interpretation and actionability of genetic variants in cardiomyopathies: a position statement from the European Society of Cardiology Council on cardiovascular genomics [PMID: 35089333].  **2018** Reappraisal of Reported Genes for Sudden Arrhythmic Death Evidence-Based Evaluation of Gene Validity for Brugada Syndrome [PMID: 29959160].  **2013** HRS/EHRA/APHRS Expert Consensus Statement on the Diagnosis and Management of Patients with Inherited Primary Arrhythmia Syndromes [PMID: 24011539]. |
| **Genetic summary** |
| **Genetic basis:** Caused by loss-of-function variants in the *SCN5A* gene, which encodes the alpha subunit of the main cardiac sodium channel Nav1.5. Several genes have been implicated in BrS, however, at present, *SCN5A* is the only definitive gene [PMID: 29959160].  **Key genes and mode of inheritance:** Please refer to **Table 1.**  **Genotype-phenotype:** Not applicable (single gene disorder).  **Disease-specific variant interpretation guidelines:** None currently available. |
| **Differential diagnoses** |
| Other inherited arrhythmia syndromes can present with symptoms which overlap with BrS (e.g., early repolarization syndrome (ERS), progressive cardiac conduction disease (PCCD), ventricular arrhythmias and syncope or SCD). However, at present, in individuals with a clinical diagnosis of BrS, genetic testing is recommended for the *SCN5A* gene only. |
| **Please note:** *This disease-specific summary is provided as an appendix to the main best practice guideline. Users should refer to the main document for recommendations on analysis, interpretation and reporting in inherited cardiomyopathies and arrhythmias.*  *The information in this appendix is based on current evidence and existing clinical and variant interpretation guidelines.*  *The core gene list (****Table 1****) takes into consideration existing ClinGen gene-disease clinical validity curations and may change as data from ongoing curation efforts becomes available.* |

| **Gene** | **Transcripts** | | **Predominant type of pathogenic variant** | **Inheritance** | **Diagnostic Yield** |
| --- | --- | --- | --- | --- | --- |
| *SCN5A* | ENST00000423572.7**^a^**  ENST00000413689.6**^b^** | NM_00335.5 **^a^**  NM_001099404.2**^b^** | Truncating; Non-truncating | AD | 10-30% |
| **Table 1.** Listed here is the ‘Definitive’ gene recommended for inclusion in Brugada Syndrome test panels. [PMID: 29959160]. **Transcripts:** Preferred transcripts for defining the regions of interest (ROI) for variant calling, and for variant annotation. Listed are the Ensembl (<https://www.ensembl.org>) and RefSeq (<https://www.ncbi.nlm.nih.gov/refseq/>) transcripts specified by the MANE collaboration (v0.95) [PMID: 35388217]. In most clinical laboratories, the ROI includes the coding regions and adjacent intronic sequence (e.g.  ± 10 to 20 nucleotides) of these transcripts. **Predominant type of pathogenic variant:** 'Truncating' includes out-of-frame insertions/deletions; variants leading to aberrant splicing (canonical splice sites or other 'deep' intronic variant); nonsense variants; copy number variants (CNVs) involving deletion of multiple exons within a gene or a whole gene. 'Non-truncating' includes missense variants and small in-frame insertions/deletions.  **Inheritance:** AD=autosomal dominant. **Diagnostic yield:**  *Approximate* proportion of probands referred for BrS testing in whom ‘likely pathogenic’, or ‘pathogenic variants’ are detected.  PMID =PubMed ID <https://pubmed.ncbi.nlm.nih.gov/>. **Note^a^**: *SCN5A* adult isoform, exon 6. **Note^b^:** *SCN5A* fetal isoform, with alternative exon 6 (denoted exon 6a). | | | | | |

| **Appendix 6: Catecholaminergic Polymorphic Ventricular Tachycardia (CPVT)** |
| --- |
| **Clinical summary** |
| **Key clinical features:** Recurrent syncope, seizures, or sudden death during physical activity or emotional stress. Normal resting electrocardiogram (ECG), exercise-induced ventricular arrhythmias (most commonly bi-directional and polymorphic ventricular tachycardia).  **Variable expressivity:** Signs and symptoms can be variable, even in relatives with the same pathogenic variant.  **Age-related and incomplete penetrance:** Typically manifests in 1st or 2nd decade but can appear at any age. Not all individuals with a pathogenic variant develop signs or symptoms, even at advanced age.  **Disease prevalence estimate/s:** ~ 1 in 10,000 [PMID: 27180891].  **Clinical and genetic testing guidelines:**  **2022** EHRA/HRS/APHRS/LAHRS Expert consensus statement on the State of Genetic Testing for Cardiac Disease [PMID:3590533].  **2022** Interpretation and actionability of genetic variants in cardiomyopathies: a position statement from the European Society of Cardiology Council on cardiovascular genomics [PMID: 35089333].  **2021** Evaluation of gene validity for CPVT and short QT syndrome in sudden arrhythmic death [PMID:34557911].  **2013** HRS/EHRA/APHRS Expert Consensus Statement on the Diagnosis and Management of Patients with Inherited Primary Arrhythmia Syndromes [PMID: 24011539]. |
| **Genetic summary** |
| **Genetic basis:** Predominantly caused by pathogenic variants in genes encoding proteins involved in regulating calcium release from the sarcoplasmic reticulum [PMID:32115705].  **Key genes and mode of inheritance:** Please refer to **Table 1.**  **Genotype-phenotype:** No gene-specific correlations have been described.  **Disease-specific variant interpretation guidelines:** None currently available. |
| **Differential diagnoses** |
| Clinical features of both CPVT and long QT syndrome have been described for some genes (*TRDN*, *TECRL*, *CALM1*, *CALM2*, *CALM3*). |
| **Please note:** *This disease-specific summary is provided as an appendix to the main best practice guideline. Users should refer to the main document for recommendations on analysis, interpretation and reporting in inherited cardiomyopathies and arrhythmias.*  *The information in this appendix is based on current evidence and existing clinical and variant interpretation guidelines.*  *The core gene list (****Table 1****) takes into consideration existing ClinGen gene-disease clinical validity curations and may change as data from ongoing curation efforts becomes available.* |

| **Gene** | **Transcripts** | | **Predominant type of pathogenic variant** | **Inheritance** | **Diagnostic Yield** |
| --- | --- | --- | --- | --- | --- |
| **Definitive/Strong evidence genes** | | | | | |
| *RYR2* | ENST00000366574.7 | NM_001035.3 | Non-truncating | AD**^a^** | 50-60% |
| *CASQ2* | ENST00000261448.6 | NM_001232.4 | Truncating; Non-truncating | AR | 2-5% |
| *TECRL* | ENST00000381210.8 | NM_001010874.5 | Truncating; Non-truncating | AR | <5% |
| *TRDN* | ENST00000546248.5**^a^**  ENST00000542443.5**^b^** | NM_001256021.1**^b^**  NM_001256022.1^c^ | Truncating; Non-truncating | AR | <1% |
| **Moderate evidence genes** | | | | | |
| *CALM1* | ENST00000356978.9 | NM_006888.6 | Non-truncating | AD**^d^** | <1% |
| *CALM2* | ENST00000272298.12 | NM_001743.6 | Non-truncating | AD**^d^** | <1% |
| *CALM3* | ENST00000291295.14 | NM_005184.4 | Non-truncating | AD**^d^** | <1% |
| **Table 1.** Listed here are the ‘Definitive’ CPVT genes recommended for inclusion in test panels [PMID:34557911]. **Transcripts:** Preferred transcripts for defining the regions of interest (ROI) for variant calling, and for variant annotation. Listed are the Ensembl (<https://www.ensembl.org>) and RefSeq (<https://www.ncbi.nlm.nih.gov/refseq/>) transcripts specified by the MANE collaboration (v0.95) [PMID: 35388217] and transcripts containing coding regions not included in the MANE transcripts (e.g. tissue-specific exons, developmental stage-specific exons) but considered relevant to clinical testing in the context of cardiac disease. In most clinical laboratories, the ROI includes the coding regions and adjacent intronic sequence (e.g.  ± 10 to 20 nucleotides) of these transcripts. However, in genes where truncating/loss-of-function variants are pathogenic (e.g., *TRDN*), test sensitivity may be increased by extending the ROI into deeper intronic regions. **Predominant type of pathogenic variant:** 'Truncating' includes out-of-frame insertions/deletions; variants leading to aberrant splicing (canonical splice sites or other 'deep' intronic variant); nonsense variants; copy number variants (CNVs) involving deletion of multiple exons within a gene or a whole gene. 'Non-truncating' includes missense variants and small in-frame insertions/deletions. **Inheritance:** AD=autosomal dominant; AR =autosomal recessive. **Diagnostic yield:**  *Approximate* proportion of probands referred for CPVT testing in whom ‘likely pathogenic’, or ‘pathogenic variants’ are detected.  PMID =PubMed ID <https://pubmed.ncbi.nlm.nih.gov/>. **Note^a^:** High proportion of *de novo* variants [PMID: 35135837]. **Note^b^** The MANE Select transcripts for *TRDN* (ENST00000334268.9/ NM_006073.4) incorporate exons that are not highly expressed in cardiac tissue, therefore only the transcripts for the predominant cardiac isoforms are listed [PMID:32167373; PMID:33692971; PMID:10497235]. **Note ^c^:** Alternative exon 6a–containing *TRDN* transcript [PMID: 32402482]. **Note^d^:** Predominantly *de novo* variants [PMID: 31170290]. | | | | | |
